# Comparison of the accident process, radioactivity release and ground contamination between Chernobyl and Fukushima-1

**DOI:** 10.1093/jrr/rrv074

**Published:** 2015-11-14

**Authors:** Tetsuji Imanaka, Gohei Hayashi, Satoru Endo

**Affiliations:** 1 Research Reactor Institute, Kyoto University, 2 Asashiro-Nishi, Kumatori-cho, Sennan-gun, Osaka 590–0494, Japan; 2 Institute of Development, Aging and Cancer, Tohoku University, Seiryo-machi 4-1, Aoba-ku Sendai, 980–8575, Japan; 3 Quantum Energy Applications, Graduate School of Engineering, Hiroshima University, 1-4-1 Kagamiyama, Higashi-Hiroshima 739–8527, Japan

**Keywords:** Fukushima accident, Chernobyl accident, ground contamination, radiation exposure, ^137^Cesium, ^131^Iodine

## Abstract

In this report, we have reviewed the basic features of the accident processes and radioactivity releases that occurred in the Chernobyl accident (1986) and in the Fukushima-1 accident (2011). The Chernobyl accident was a power-surge accident that was caused by a failure of control of a fission chain reaction, which instantaneously destroyed the reactor and building, whereas the Fukushima-1 accident was a loss-of-coolant accident in which the reactor cores of three units were melted by decay heat after losing the electricity supply. Although the quantity of radioactive noble gases released from Fukushima-1 exceeded the amount released from Chernobyl, the size of land area severely contaminated by ^137^Cesium (^137^Cs) was 10 times smaller around Fukushima-1 compared with around Chernobyl. The differences in the accident process are reflected in the composition of the discharged radioactivity as well as in the composition of the ground contamination. Volatile radionuclides (such as ^132^Te-^132^I, ^131^I, ^134^Cs and ^137^Cs) contributed to the gamma-ray exposure from the ground contamination around Fukishima-1, whereas a greater variety of radionuclides contributed significantly around Chernobyl. When radioactivity deposition occurred, the radiation exposure rate near Chernobyl is estimated to have been 770 μGy h^−1^ per initial ^137^Cs deposition of 1000 kBq m^−2^, whereas it was 100 μGy h^−1^ around Fukushima-1. Estimates of the cumulative exposure for 30 years are 970 and 570 mGy per initial deposition of 1000 kBq m^−2^ for Chernobyl and Fukusima-1, respectively. Of these exposures, 49 and 98% were contributed by radiocesiums (^134^Cs + ^137^Cs) around Chernobyl and Fukushima-1, respectively.

## INTRODUCTION

The worst-case scenarios at nuclear power plants are accidents that result in direct release of accumulated radioactivity into the environment from the reactor core [1, 2]. Two kinds of accidents that can cause such a situation have been of concern since the beginning of nuclear power development: power-surge accidents and loss-of-coolant accidents. The Chernobyl Nuclear Power Station (NPS) accident of 1986 belongs to the former group: the power surge was caused by a failure to control fission chain reactions, which led to an explosion that instantaneously destroyed the reactor, together with its building [3, 4]. The Fukushima-1 accident of 2011 belongs to the latter group: the earthquake and tsunami subsequently let to loss of both the offsite and onsite power supply, leading to reactor core meltdown in three reactors out of six units at the Fukushima-1 NPS [5, 6]. Both the Chernobyl and Fukushima-1 accidents are classified as Level-7, the worst level on the International Nuclear Event Scale (INES) of the International Atomic Energy Agency (IAEA).

In order to investigate the radiological impacts on biota from nuclear accidents, it is important to obtain detailed information about the composition of the radioactivity contamination and about the level of radiation exposure in the environment. In this paper, the basic features of the accident processes and the radiological consequences (such as radioactivity release into the atmosphere, ground contamination and gamma-ray exposure above the ground) are compared between Chernobyl and Fukushima-1.

### Chernobyl accident

The Chernobyl-type reactor (RBMK-1000, 1000 MWe) was developed by the former USSR based on the reactor for producing plutonium for nuclear weapons, and it was only used inside USSR territory. At the time of the Chernobyl accident in April 1986, there were 15 RBMK reactors operating at five NPSs in the USSR. At the Chernobyl NPS in the Ukraine, four RBMK-1000 reactors were operating and two others were under construction. From the structure of the reactor, RBMK can be classified as a graphite-moderator, boiling light-water cooling and channel-type reactor, which have the following weaknesses:

- a positive void reactivity coefficient that appears when the steam fraction increases in the fuel channels;
- a ‘positive scram’ effect when all control rods are inserted into the core at the same time under certain extreme operation conditions;
- complexity of reactor control, due to a large number of channels in the core.

At midnight 24 April 1986, operation staff at the Chernobyl-4 unit (3200 MWt) began to prepare the reactor for shutdown for maintenance for the first time since the start of operation in December 1983. During the process of the shutting down the reactor, several tests were planned, including testing of a new emergency generator system using the inertial energy of the freewheeling turbines post shutdown. Although this test was scheduled for during the day on 25 April at a power level of 700–1000 MWt, it was postponed till the midnight. At around 00:30 on 26 April, the reactor power suddenly fell to almost zero. The operators tried to revive the reactor power, by pulling out almost all control rods from the reactor core. At around 01:00, when the reactor was stabilized at a power level of 200 MWt, it was decided to carry out the generator test at a power level less than that planned.

At 01:23:04, by closing the steam valve to the turbine, the generator test started. An emergency event started at 01:23:40 when the operators turned on the AZ-5 button to shut down the reactor by inserting all control rods into the core. Contrary to the intention of the operator, a positive scram phenomenon led to a small power surge in the lower part of the core, damaging the nuclear fuels and channel tubes. Following rupture of the channel tubes, a large amount of water vapor appeared at the core. Then, a bigger-scale power surge was caused by the effect of the positive void coefficient of reactivity, which led an explosion of the reactor and destruction of the building. According to the analysis after the accident, the explosion is believed to have occurred 6–7 s after turning on AZ-5. Eyewitnesses outside the reactor building said that there was a series of explosion-like fireworks reaching up into the night sky. (The above accident process is summarized from references [3] and [4].)

**Fig. 1.**
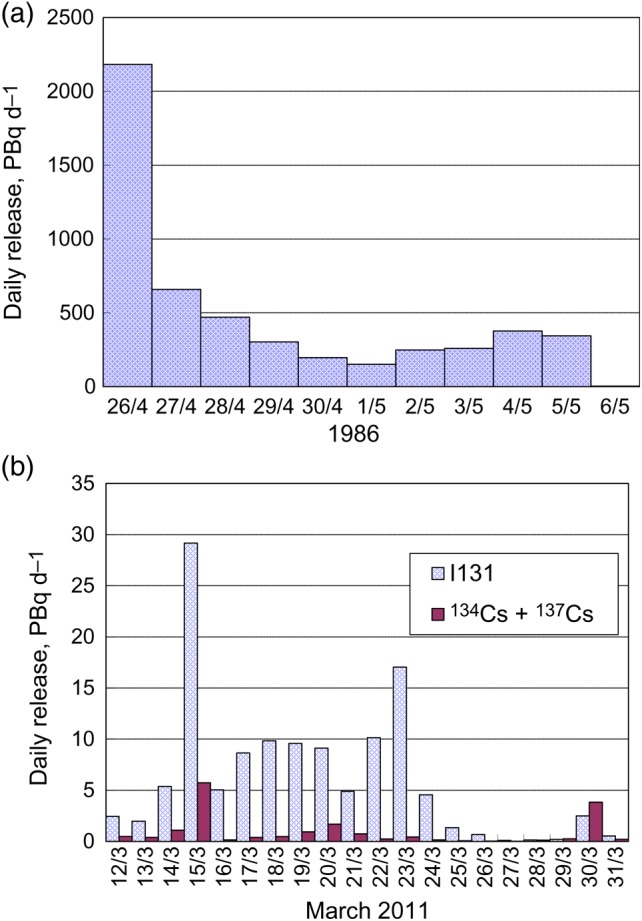
Estimates of daily release of radioactivity into the atmosphere. (**a**) Chernobyl. Values are for all radionuclides except rare gases and are decay-corrected to 26 April 1986. (**b**) Fukushima-1. Daily release values are calculated by the current authors based on the hourly data in ref. [7].

Graphite in the reactor core began to burn after the initial explosion. This fire continued for more than ten days, releasing a large amount of the radioactivity that had accumulated in the core. The daily discharge of radioactivity (based on estimates in the 1986 USSR report [3]) is shown in Fig. 1a.

### Fukushima-1 accident

There were six boiling water reactor (BWR) units on the site of the Fukushima-1 NPS (Unit 1: 460 MWe; Units 2–5: 784 MWe; Unit 6: 1100 MWe). When the earthquake occurred at 14:46 on 11 March 2011, three units (Unit 1, Unit 2 and Unit 3) were operating in full power, and the remaining three were out of operation due to annual maintenance.

On the arrival of seismic waves, all three operating units were successfully tripped by automatic insertion of control rods. However, the transmission line for the offsite power supply was broken due to a transmission tower falling down, as well as transformer damages at the substation. In order to avoid blackout of the NPS, an Emergency Diesel Generator (EDG) was automatically actuated at each unit. About 40 min later, a series of tsunami waves hit the Fukushima-1 NPS, with wave heights of more than 10 m. The anti-tsunami protection was designed for ∼6 m. All EDGs from Unit 1 to Unit 4 were located in the basement of the turbine buildings and flooded with seawater. Consequently, AC power was lost for cooling the reactor cores of Unit 1 to Unit 3. In addition, the DC batteries also became unavailable in Unit 1 and Unit 2 for powering the process instruments and control valves.

When the cooling system does not work after shutdown of the reactor, decay heat from fission products in the core will cause increase in both the temperature and the pressure of the coolant water, which will eventually result in meltdown of the reactor core and melt-through of the reactor vessel. In order to avoid such a situation, several emergency cooling systems working without AC power were installed in the BWR: an Isolation Condenser (IC) in Unit 1, a Reactor Core Isolation Cooling (RCIC) system in Units 2 and 3 and a High Pressure Core Injection (HPCI) system in Units 1 to 3. In reality, however, these emergency systems could not prevent further development of the situation, partly because the DC power necessary to actuate and control them was lost at Units 1 and 2, and partly because they were not designed to be effective for such a long period as was needed during the event in Fukushima-1.

At Unit 1, its reactor core began to be damaged in the evening of 11 March, and a high Containment Vessel (CV) pressure of 840 kPa was recorded at midnight, which was about two times higher than the pressure for which it was designed (427 kPa). It was clear that CV destruction at this stage would mean the worst situation at the NPS. Around 14:30 on 12 March, CV venting was finally carried out to reduce the CV pressure. Then at 15:36 a hydrogen explosion occurred inside the upper part of the reactor building. Core cooling of Unit 1 by fire engines started in the evening of 12 March.

At Unit 3, after the tsunami hit, core cooling was maintained by RCIC until 11:36 on 12 March. After RCIC stopped, HPCI was automatically actuated and continued working until 02:42 on 13 March. Its core damage is believed to have begun on the morning of 13 March. PC venting was carried out several times on 13 March. At 11:01 on 14 March, a hydrogen explosion occurred inside the reactor building.

At Unit 2 at the time the tsunami hit, the RCIC was working. It continued to work without DC power until 13:25 on 14 March. Its core damage is believed to have begun on the evening of 14 March. CV venting was tried but was unsuccessful. A high CV pressure of 600 kPa was recorded during the night and this high pressure continued until the next morning. A rapid decrease in the CV pressure was observed at about 06:00 on 15 March, which indicated serious damage to the CV integrity of Unit 2. This resulted in the largest radioactivity release that occurred during the course of the Fukushima-1 accident.

The daily radioactivity discharge of ^131^I and ^134^Cs + ^137^Cs from the Fukushima-1 NPS is shown in Fig. 1b, based on estimates by UNSCEAR [7].

### Radioactivity release

The radioactivity released into the atmosphere for Chernobyl and Fukushima-1 is compared in Table 1. The estimates of the Chernobyl Forum [8] are a summary from various studies.

**Table 1.** Estimates of released radioactivity (PBq) of major radionuclides into the atmosphere: Chernobyl and Fukushima-1

	Chernobyl [8]	Fukushima-1 [7]
^133^Xe	6500	7300
^131^I	1760	120
^132^Te	1150	29
^134^Cs	47	9.0
^137^Cs	85	8.8
^90^Sr	10	n.a.
^95^Zr	84	n.a.
^103^Ru	168	n.a.
^106^Ru	73	n.a.
^140^Ba	240	n.a.
^141^Ce	84	n.a.
^239^Np	400	n.a.
^239^Pu	0.013	n.a.

n.a. = not assessed.

The UNSCEAR values [7] are mainly based on Terada *et al*. [9], which report itself is based on an inversion technique combining the monitoring data from the environment with the results of atmospheric transport simulation of released radioactivity.

It is clear that the ^131^I and ^137^Cs release from Fukushima-1 was significantly less than from Chernobyl. Comparison of the findings of UNSCEAR and the Chernobyl Forum indicates that ^131^I and ^137^Cs releases from Fukushima-1 were 7% and 10% of the respective releases from Chernobyl. The released radioactivity in the form of ^90^Sr, ^239^Pu and other radionuclides from Fukushima-1 is considered to be far less than that released from Chernobyl, which reflects the difference in the accident process. In the case of the Chernobyl accident, the explosion occurred inside the reactor core, and the reactor materials themselves (such as nuclear fuels and graphite blocks) were dispersed into the atmosphere. Thus, the composition of the radionuclides discharged from Chernobyl was similar to that found in the reactor core. In contrast to this, the reactor cores did not explode in Fukushima-1, and the radioactivity discharge was mostly composed of gaseous and volatile radionuclides emitted from the damaged and melted reactor cores. Two hydrogen explosions occurred at Fukushima-1 under the roof of the reactor building of Unit 1 and Unit 3, but they were not inside the CVs.

Far less discharge of ^90^Sr and ^239,240^Pu into the atmosphere from Fukushima-1 than Chernobyl was confirmed by the measurement of soil samples. Table 2 shows ^90^Sr, ^239,240^Pu and ^137^Cs contamination in soil samples taken in Iitate village [10] together with those taken in Kiev [11]. Deposition ratios of ^90^Sr and ^239,240^Pu, to ^137^Cs were 0.23 and 0.006, respectively, in Kiev whereas they were 0.0004 – 0.0005 and 10^−7^– 10^−8^ in Iitate.

**Table 2.** Comparison of ^137^Cs, ^90^Sr and ^239,240^Pu contamination in the soil of Iitate village and Kiev city

	Contamination density, Bq m^−2^		
	^137^Cs	^90^Sr	^239,240^Pu
Iitate village: 30–40 km north-west of Fukushima-1 NPS [10]			
Sample 1	1 000 000	390^a^	0.03
Sample 2	590 000	300^a^	0.07
Sample 3	2 200 000	790^a^	0.2
Kiev city: 110 km South of Chernobyl NPS [11]			
Average of six samples	25 000	5800	160

^a^Measured by the Kyushu Environmental Evaluation Association. Values include global fallout.

### Ground contamination and radiation exposure

In regard to the long-term effects of radioactive contamination in the environment, ^137^Cs is the most important radionuclide, both in Chernobyl and Fukushima-1. The size of area severely contaminated by ^137^Cs for the two accidents is compared in Table 3 [12, 13]. The contaminated area around Chernobyl is more than 10 times larger than Fukushima-1. It is noteworthy, however, that although the Chernobyl NPS is surrounded by land, the eastern half of the surroundings of Fukushima-1 is in the Pacific Ocean, and most of the discharged radioactivity from Fukushima-1 is believed to have streamed toward the ocean, blown by the prevailing westerlies over Japan.

**Table 3.** Size of severely contaminated area around Chernobyl and Fukushima-1

	^137^Cs contamination level	
	From 555 to 1480 kBq m^−2^	>1480 kBq m^−2^
Chernobyl [12]	7200 km^2^	3100 km^2^
Fukushima-1 [13]	495 km^2^	272 km^2^

^137^Cs contamination level of 555 and 1480 kBq m^−2^ corresponds to criteria for compulsory resettlement and alienation, respectively, around Chernobyl.

The radionuclide composition of the ground contamination within 100 km of the Chernobyl NPS is reported by Izrael *et al.* [14]. They indicate that the composition varies, depending on direction from the NPS and also on distance. In Table 4, the relative deposition ratios of major radionuclides to ^137^Cs contributing gamma-ray exposure are shown for the near western area of the Chernobyl NPS, where the initial plume passed over on the first day of the accident. The relative deposition ratios around Fukushima-1 are also shown in Table 4 (values are taken from UNSCEAR [7]).

**Table 4.** Deposition ratios of major radionuclides to ^137^Cs that contributed gamma-ray exposure at 1 m above ground

Radionuclide	Half-life	Exposure rate conversion factor (nGy h^−1^)/ (kBq m^−2^)	Relative deposition ratio to ^137^Cs	
			Chernobyl [14]	Fukushima-1 [7]
^95^Zr	65.5 d	2.82	20	
^95^Nb	35.0 d	2.92	20	
^103^Ru	39.3 d	1.85	16	
^131^I	8.04 d	1.49	18	11.5
^132^Te	3.25 d	0.79	28	8
^132^I	(2.30 h)^a^	8.61	28	8
^134^Cs	2.07 y	5.97	0.4	1
^137^Cs	30.1 y	2.18	1	1
^140^Ba	12.8 d	0.57	22	
^140^La	(1.68 d)^a^	7.83	11	
^239^Np	2.36 d	0.60	120	

^a^These radionuclides are treated at radioactive equilibrium with parent radionuclides. Exposure rate conversion factors are taken from Beck [15] for a case of 0.16 g cm^−2^ of relaxation length for depth distribution. Values for Chernobyl are values on the day of the accident, 26 April 1986. Values for Fukushima-1 are on 15 March 2011, when the most severe ground contamination occurred.

**Fig. 2.**
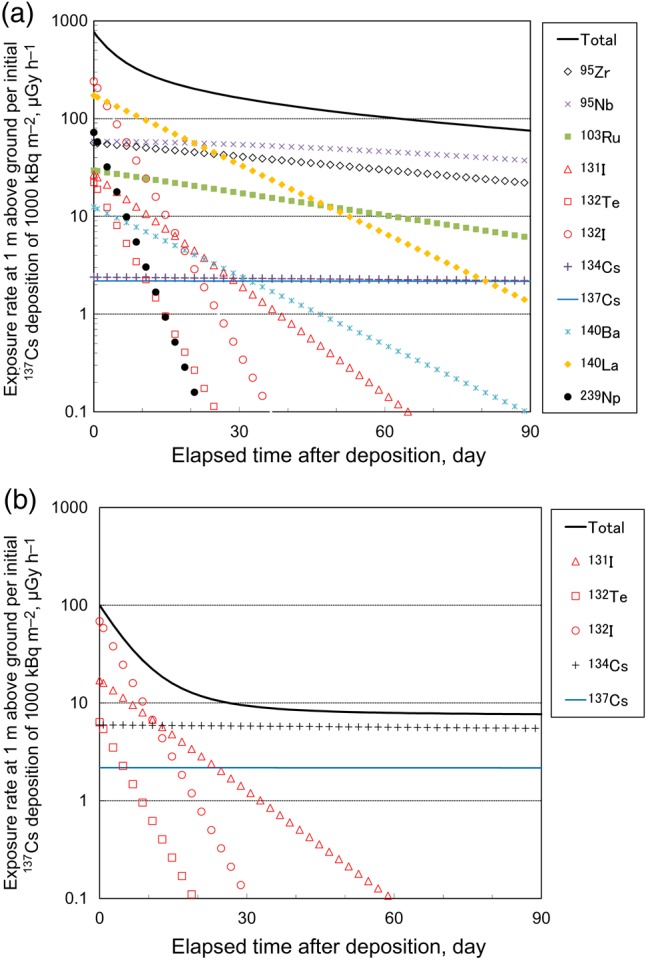
Temporary change of gamma-ray exposure rate at 1 m above ground per initial ^137^Cs deposition of 1000 kBq m^−2^ for 90 days after the deposition. (a) Chernobyl. Relative deposition ratios to ^137^Cs are taken from Izrael et al [14] for near western area from Chernobyl NPS. (b) Fukushima-1. Deposition ratios are taken from UNSCEAR [7] for all Japan except southern direction from Fukushima-1 NPS.

Based on the deposition ratios in Table 4, the gamma-ray exposure rates at 1 m above ground per initial ^137^Cs deposition of 1000 Bq m^−2^ are calculated and plotted in Fig. 2 for 90 days after deposition. Radiation exposure conversion factors are taken from Beck [15] for a case of 0.16 g cm^−2^ of relaxation length as depth distribution. A clear difference is seen between Chernobyl and Fukushima-1. Compared with Fukushima-1, ^95^Z, ^95^Nb, ^103^Ru and ^140^La made a significant contribution to the radiation exposure resulting from the Chernobyl accident. The initial exposure rate of 770 μGy h^−1^ for Chernobyl was 7.7 times larger than that for Fukushima-1, which was 100 μGy h^−1^. After 30 days, almost all the radiation exposure for Fukushima-1 was due to radiocesiums (^134^Cs and ^137^Cs), whereas the contribution of radiocesiums for Chernobyl was only 6% of the total exposure, even on the 90th day.

**Fig. 3.**
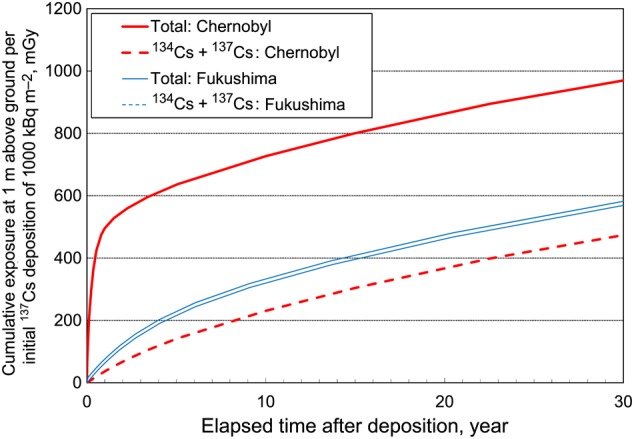
Cumulative gamma-ray exposure at 1 m above ground per initial ^137^Cs deposition of 1000 kBq m^−2^ up to 30 years after the deposition: Chernobyl and Fukushima-1. Solid lines indicate total exposure and dashed lines are sum of contribution from ^134^Cs and ^137^Cs. Thick lines and thin lines are for Chernobyl and Fukushima-1, respectively.

The cumulative exposure normalized to the initial ^137^Cs deposition of 1000 kBq m^−2^ is shown in Fig. 3 for the first 30 years after deposition, both for Chernobyl and Fukushima-1. Thick solid and thick dashed lines indicate the total exposure and the sum of the contributions from ^134^Cs and ^137^Cs, respectively, for Chernobyl. Thin solid and thin dashed lines indicate the equivalent values for Fukushima-1. The total cumulative exposure for the first year and the first 30 years are 500 and 970 mGy, respectively, for Chernobyl, whereas the values are 63 and 570 mGy for Fukushima-1. The difference in total radiation exposure between Chernobyl and Fukushima is mainly due to the exposure during the first year when ^95^Z, ^95^Nb, ^103^Ru and ^140^La made a significant contribution. The contribution of radiocesiums to the total exposure for the first year was 7.4 and 83% for Chernobyl and Fukushima-1, respectively. For the period of 30 years the contributions have been calculated to be 49 and 98% for Chernobyl and Fukushima-1, respectively.

From the point of long-term impact on the environment by the Fukushima-1 accident, our attention should be focused on radiation exposure from radiocesiums. The situation around Chernobyl is different from that around Fukushima-1 because radionuclides other than radiocesiums (e.g. ^90^Sr and Pu isotopes) are believed to make a greater contribution at Chernobyl. We note that radioactive contamination in the Pacific Ocean by the Fukushima-1 accident has not been discussed here. Several studies on radioactivity release into the Pacific Ocean have been summarized by UNSCEAR [7], but there are large areas of uncertainty. Considering the leakage of contaminated water continues to flow through an unknown underground path from the Unit buildings, more efforts are needed for study of the radioactivity release into the Pacific Ocean.

## CONCLUSION

The Chernobyl accident was a power-surge accident in which failure to control a fission chain reaction instantaneously destroyed the reactor and building, whereas the Fukushima-1 accident was a loss-of-coolant accident in which the reactor cores of three units were melted by decay heat after the loss of electricity. The differences in the accident processes are reflected in the composition of the ground contamination. Only volatile radionuclides (such as ^132^Te-^132^I, ^131^I, ^134^Cs and ^137^Cs) contributed gamma-ray exposure around Fukushima-1, but a variety of radionuclides contributed significantly around Chernobyl. At the time when the radioactivity deposition occurred, the radiation exposure rate near Chernobyl is estimated to have been 770 μGy h^−1^ per initial ^137^Cs deposition of 1000 kBq m^−2^, whereas it was 100 μGy h^−1^ around Fukushima-1. Estimates of the cumulative exposure for 30 years are 970 and 570 mGy per initial deposition of 1000 kBq m^−2^ for Chernobyl and Fukushima-1, respectively. Of these exposures, 49 and 98% were contributed by radiocesiums (^134^Cs + ^137^Cs) around Chernobyl and Fukushima-1, respectively.

## FUNDING

This work was supported by the Japan Society for the Promotion of Science (JSPS) [KAKENHI Grant No. 26301003]. Funding to pay the Open Access publication charges for this special issue was provided by the Grant-in-Aid from the Japan Society for the Promotion of Science (JSPS) [KAKENHI Grant No. 26253022].
